# Study of GABA in Healthy Volunteers: Pharmacokinetics and Pharmacodynamics

**DOI:** 10.3389/fphar.2015.00260

**Published:** 2015-11-10

**Authors:** Junfeng Li, Zhaoyun Zhang, Xiaoxia Liu, Yi Wang, Fei Mao, Junjun Mao, Xiaolan Lu, Dongdong Jiang, Yun Wan, Jia-Ying Lv, Guoying Cao, Jing Zhang, Naiqing Zhao, Mark Atkinson, Dale L. Greiner, Gerald J. Prud'homme, Zheng Jiao, Yiming Li, Qinghua Wang

**Affiliations:** ^1^Department of Endocrinology and Metabolism, Huashan Hospital, Fudan UniversityShanghai, China; ^2^Department of Pharmacy, Huashan Hospital, Fudan UniversityShanghai, China; ^3^Department of Biostatistics, School of Public Health, Fudan UniversityShanghai, China; ^4^Key Laboratory of Clinical Pharmacology of Antibiotics, Ministry of Health, Institute of Antibiotics, Huashan Hospital, Fudan UniversityShanghai, China; ^5^Department of Pathology, College of Medicine, University of FloridaGainesville, FL, USA; ^6^Department of Molecular Medicine, University of Massachusetts Medical SchoolWorcester, MA, USA; ^7^Department of Laboratory Medicine and Pathobiology, Keenan Research Centre for Biomedical Science of St. Michael's Hospital, University of TorontoToronto, ON, Canada; ^8^Division of Endocrinology and Metabolism, The Keenan Research Centre in the Li Ka Shing Knowledge Institute, St. Michael's HospitalToronto, ON, Canada; ^9^Department of Physiology and Medicine, University of TorontoON, Canada

**Keywords:** GABA, pharmacokinetics, glucagon, insulin, glycated albumin

## Abstract

Preclinical studies show that GABA exerts anti-diabetic effects in rodent models of type 1 diabetes. Because little is known about its absorption and effects in humans, we investigated the pharmacokinetics and pharmacodynamics of GABA in healthy volunteers. Twelve subjects were subjected to an open-labeled, three-period trial involving sequential oral administration of placebo, 2 g GABA once, and 2 g GABA three times/day for 7 days, with a 7-day washout between each period. GABA was rapidly absorbed (Tmax: 0.5 ~ 1 h) with the half-life (t1/2) of 5 h. No accumulation was observed after repeated oral GABA administration for 7 days. Remarkably, GABA significantly increased circulating insulin levels in the subjects under either fasting (1.6-fold, single dose; 2.0-fold, repeated dose; *p* < 0.01) or fed conditions (1.4-fold, single dose; 1.6-fold, repeated dose; *p* < 0.01). GABA also increased glucagon levels only under fasting conditions (1.3-fold, single dose, *p* < 0.05; 1.5-fold, repeated dose, *p* < 0.01). However, there were no significant differences in the insulin-to-glucagon ratio and no significant change in glucose levels in these healthy subjects during the study period. Importantly, GABA significantly decreased glycated albumin levels in the repeated dosing period. Subjects with repeated dosing showed an elevated incidence of minor adverse events in comparison to placebo or the single dosing period, most notably transient discomforts such as dizziness and sore throat. However, there were no serious adverse events observed throughout the study. Our data show that GABA is rapidly absorbed and tolerated in human beings; its endocrine effects, exemplified by increasing islet hormonal secretion, suggest potential therapeutic benefits for diabetes.

## Introduction

Type 1 diabetes (T1D) is an autoimmune disease, characterized by progressive loss of functional β-cell mass, resulting from insulitis, which leads to insulin deficiency, elevation of blood glucose, and various complications associated with this disease (Atkinson et al., 2014). An ideal effective therapy may require two arms: β-cell regeneration and immunosuppressive effects. However, none of the current therapies effectively achieve both goals (Lernmark and Larsson, 2013; Atkinson et al., 2014).

Recently we and others have shown that gamma aminobutyric acid (GABA) exerts β-cell regenerative and immunoregulatory effects (Soltani et al., 2011; Prud'homme et al., 2013; Tian et al., 2013; Purwana et al., 2014). Specifically, GABA stimulates β-cell replication, protects β-cells against apoptosis, and attenuates insulitis (Soltani et al., 2011; Tian et al., 2013; Prud'homme et al., 2014; Purwana et al., 2014). These effects result in an enhanced functional β-cell mass and, in mice, this can reverse disease (Soltani et al., 2011). These favorable effects were first observed in mice (Tian et al., 2004, 2014), but appear valid in humans as demonstrated *in vitro* as well as in xenotransplanted human islets (Tian et al., 2013; Purwana et al., 2014).

GABA, identified initially in the central nervous system, is produced by pancreatic β-cells in large quantities (Adeghate and Ponery, 2002). While acting as an inhibitory neurotransmitter in the adult brain (Owens and Kriegstein, 2002), GABA exerts excitatory trophic effects such as neuronal cell proliferation and dendritic maturation in the developing brain (Represa and Ben-Ari, 2005). GABA exerts its biological effects through the activation of GABA receptors that are expressed in a variety of peripheral tissues, and cells including pancreatic islet cells and immune cells such as T and B lymphocytes (Tian et al., 2011). Within an islet, GABA suppresses glucagon secretion by the α-cells as a consequence of membrane hyperpolarization (Rorsman et al., 1989; Braun et al., 2004) but, in contrast, it enhances insulin secretion by the β-cells through membrane depolarization (Rorsman et al., 1989; Dong et al., 2006; Bansal and Wang, 2008).

In some Western countries, GABA is an amino acid health care product and used as an added component of various foods or a nonprescription drug, for various indications such as sleep or anxiety disorders; and studies using GABA supplementation in healthy individuals following daily GABA intakes up to 18 g for 4 days or 120 mg for 12 months indicated that GABA was well tolerated (Cavagnini et al., 1980; Abdou et al., 2006; Yoto et al., 2012)^1^. In China, GABA, which is listed in the Chinese Pharmacopeia [National Drug Standards, Drug Standards No. WS-10001-(HD-0871)-2002], has been clinically used for other indications such as hepatic coma rather than diabetes more than decades.

Although GABA is administered in grams per day according to drug label approved by China FDA, there is little information available in the public domain with respect of GABA's pharmacokinetics (PK) and pharmacodynamics (PD) in humans. In order to establish a GABA PK and PD profile in humans which may facilitate researchers' efforts to exam whether GABA is effective of diabetes in humans, we conducted a phase 1 clinical study which was open-labeled, three period, sequential study in 12 healthy subjects. The following oral treatments were applied: placebo, single dose of 2 g GABA, or repeated dose of 2 g GABA three times daily for 7 days, with a 7-day washout between each period to evaluate the PK, PD, and safety profile.

## Materials and methods

### Subjects

A total of 12 healthy volunteers were recruited (6 male, 6 female, aged 26 ± 1 years, BMI 22 ± 0.5 Kg/m^2^; body weight 61.2 ± 2.2 Kg). One volunteer withdrew before the single dose period. All subjects were not on any medication 2 weeks prior to screening and had no blood donation within 3 months before screening. The exclusion criteria were: abnormalities of physical examination, laboratory tests, or electrocardiogram (ECG) in screening, which may influence the results of the study; previous or existing history of severe heart, liver, kidney, gastrointestinal, nervous system, mental, or metabolic abnormalities, and other diseases which can affect drug absorption, circulation, metabolism, or excretion; history of alcoholism, smoking, or drug abuse within the past 1 year; participation in any clinical drug study within the past 30 days; any definite or suspected allergy or family history of allergy to GABA or any other similar drugs.

### Study overview

This was a single-center, open-labeled, three-period (with fixed sequence of assessments: placebo, single dose, repeated dose), and self-controlled study (Supplementary Table 1). In period 1 (placebo period), blood was sampled on day 1 (2 g placebo tablet dosing once at 8:00 AM) to obtain the baseline values. In period 2 (single dose period), blood was sampled on day 8 (2 g GABA tablet dosing once at 8:00 AM). In period 3 (repeated dose period), blood was sampled on day 22 [2 g GABA tablet dosing three times per day (8:00 AM, 12:00 AM, and 6:00 PM prior to meals) from day 15 to day 21, then 2 g GABA tablet dosing last time at 8:00 AM on day 22]. There were 7 days wash-out intervals between two periods. Subjects were hospitalized for the entire repeated-dose period, and had standardized food intake to avoid eating foods known to contain high quantities of GABA (e.g., potatoes, soybeans) during the study. The GABA (or placebo) tablets were from Shanghai Xinyi Pharmaceuticals CO., Ltd.

In each period, blood were sampled at pre-dose, 0.25, 0.5, 0.75, 1, 1.5, 2, 3, 4, 6, 8, 10, 12, and 24 h post-dose for PK and PD study. Other laboratory tests are listed in Supplementary Table 1. All these blood samples were collected from forearm vein.

This study was approved by Institutional Review Board of Huashan Hospital, and complied with the Declaration of Helsinki. All volunteers signed the informed consent. The study was conducted at the Phase1 Unit, Huashan Clinical Trial Base (approved and certificated by China State Food and Drug Administration).

This trial is registered with ClinicalTrials.gov, number NCT01917760.

### Safety assessments

In each period, before 8:00 AM, subjects underwent physical examination, 12-lead ECG, and detection of any adverse events using open questions. Vital signs (blood pressure and heart rate) and open questions for detecting adverse events were performed at pre-dose, 4, and 12 h post-dose. Participants could also report adverse events anytime during the study. At 24 h post-dose, the participants were required to undergo safety analysis including blood/urine routine tests, chemistry test, 12-lead ECG and monitoring of adverse events. According to U.S. FDA, any event which causes death, permanent damage, birth defects, or requires hospitalization is defined as serious adverse event.

### Pharmacokinetic assessments

Blood samples for GABA detection were collected into a precooled Vacutainer® EDTA-plasma tubes at pre-dose and 0.25, 0.5, 0.75, 1, 1.5, 2, 3, 4, 6, 8, 10, 12, and 24 h after dosing on day 1, 8, and 22. Within 3 min, plasma was prepared by centrifugation (3000 g at 4°C for 10 min) and stored at −80°C until analysis.

The determination of GABA levels was accomplished by the Shanghai Center for Drug Metabolism and Pharmacokinetics Research, Shanghai Institute of Materia Medica, Chinese Academy of Sciences (Shanghai, China).

A Shimadzu High-Performance Liquid Chromatography system LC-20AD (Shimadzu Corp., Japan) was used to perform the separation of GABA and internal standard (d_2_-GABA). The separation was achieved on a Phenomenex Luna HILIC column (100 mm × 3.0 mm, 3 μm; Phenomenex Corp., USA) maintained at 40°C. The mobile phase consisted of water–acetonitrile (20:80, v/v) at a flow rate of 0.5 mL/min. The injection volume was 5 μL.

A triple quadruple mass spectrometer (ABI 4000 II, Applied Biosystems Corp, USA) was equipped with an electrospray ionization (ESI) for analytical detection. The ESI source was set in positive ionization mode. Multiple reactions monitoring (MRM) was used to monitor precursor to product ion transition of m/z 104 → 69 for GABA, and m/z 106 → 71 for d_2_-GABA with scan time of 0.10 s per transition. The data acquisition and sample quantification were operated using Analyst 1.6 software (Applied Biosystems Corp, USA).

An aliquot of the plasma sample (100 μL) was transferred to an Eppendorf micro tube for processing. 25 μL internal standard (500 ng/mL d_2_-GABA) 100 μL, methanol:water (1:1, v/v) and 500 μL acetonitrile were added and vortex-mixed for 1 min. After centrifugation for 10 min at 13,000 rpm, a 5 μL aliquot of the supernatant was injected onto the LC–MS/MS system for analysis.

The calibration range was 5.0–1000 ng/mL in plasma. The extraction recovery of GABA at the low, middle and high level of quality control was 92.7, 91.5, and 98.2%, respectively. The precision (RSD) and accuracy (relative bias) of the method were evaluated to be within 9.9% and from −0.9 to 4.3%.

The PK parameters for GABA were calculated after substratction of baseline GABA concentration and estimated from plasma samples, by means of standard non-compartmental methods using the WinNonlin software (Version 4.1, Pharsight Corp, USA). The maximum plasma concentration (C_max_) and the corresponding time (T_max_) were determined directly from the concentration-time profiles during the three periods. The areas under the concentration-time curves from zero to 24 h (AUC_0–24h_) and to 4 h (AUC_0–4h_) were calculated using the linear trapezoidal rule. Other PK parameters assessed included oral clearance (CL/F), apparent volume of distribution (V/F), ratio of accumulation (RA), area under the curve from the time zero extrapolated to infinity (AUC_inf_).

### Pharmacodynamic assessments

#### GLP-1 and glucagon

One milliliter blood sample for GLP-1 (active form) and glucagon detection was collected into a precooled Vacutainer® EDTA-plasma tube that contained Sitagliptin (Sigma S8576, final blood concentration was 100 μmol/L) and Aprotinin (Sigma A1153, final blood concentration was 250 KIU/ml) at pre-dose, 0.25, 0.5, 1, 2, 4, 6, 8, 10, 12, and 24 h post-dose from subjects during the three periods as PK analysis. Immediately, tubes were inverted to mix followed by immediate centrifugation (3000 g at 4°C for 10 min) and storage at −80°C until analysis. Plasma glucagon concentrations were measured by ELISA (Mercodia, Catalog No. 10-1271-01). Plasma GLP-1 (active form) concentrations were also measured by ELISA (Millipore, Catalog No. EGLP-35K).

#### Insulin, c-peptide, and blood glucose

0.5 ml blood sample for insulin and c-peptide detection was collected into a Vacutainer® serum separation tube at pre-dose, 0.25, 0.5, 0.75, 1, 1.5, 2, 4, 6, 8, 10, 12, and 24 h post-dose from subjects during the three periods as PK analysis. Concurrently, 0.5 ml blood sample for blood glucose detection was collected into a Vacutainer® glucose tube that contained sodium fluoride. Serum insulin and c-peptide concentrations were measured by a electrochemiluminescence immunoassay technique (Roche Elecsys 2010). Plasma glucose levels were measured by a glucose oxidase method (Abbott C8000 analyzer).

The area under the concentration-time curve of GLP-1, glucagon, insulin, c-peptide and blood glucose levels from the time zero to 4 h (AUC_0–4h_) and to 24 h (AUC_0–24h_) were calculated using the trapezoidal rule.

#### Glycated albumin

Glycated albumin was measured by enzymatic assay on day −2, day 15, and day 23 (Asahi Kasei Corporation, Japan. Lucica™ GA-L assay kit). Day −2 was the screening day. Day 15 was the beginning day of the repeated dose period. Day 23 was the sampling day of the repeated dose period.

#### Statistical methods

Descriptive statistics of means and standard error (SE) were calculated for continuous parameters, as well as frequencies and percentages for categorical parameters. AUC were logarithmically transformed before analysis and then subjected to ANOVA. If data were not normally distributed or did not met the homogeneity of variances, nonparametric tests were used for comparisons among multiple groups. Data were processed using GraphPad Prism version 6.0 (GraphPad Software, Inc, La Jolla, CA) and Statistical Product and Service Solutions (SPSS) version 19.0 (SPSS, Inc, Chicago, IL). All tests were two-sided, with *P* < 0.05 considered statistically significant.

## Results

### Pharmacokinetic profile

**Figure 1:**
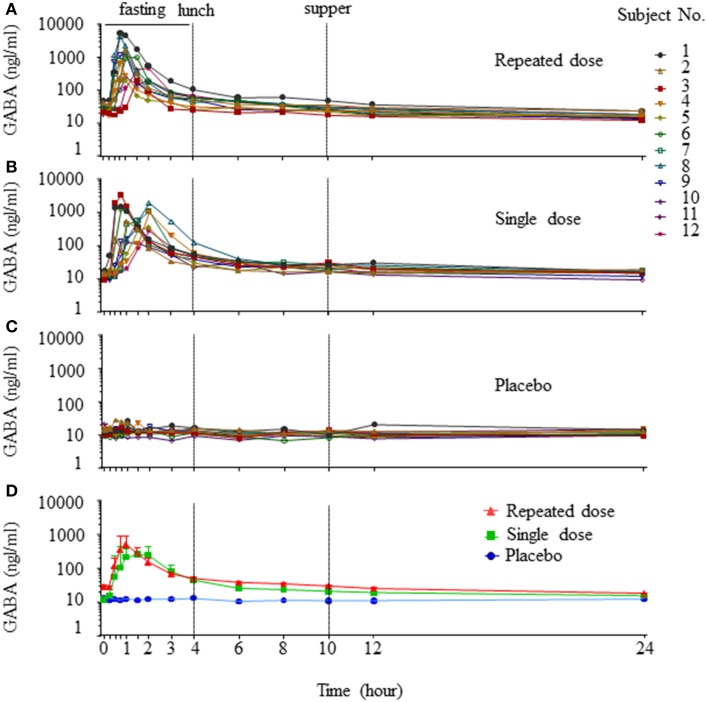
**Concentration-time profiles of GABA**. Individual concentration-time curves of GABA in the three periods (**A**, repeated dose period; **B**, single dose period; **C**, placebo period). Total concentration-time curves of GABA in the three periods, data are presented as geomean ± SE **(D)**.

Individual concentration-time profiles of GABA following oral administration in the three periods were determined (Figures 1A–C). Given the obvious variations among the study subjects in each period, data are presented as geometric mean ± SE (Figure 1D). When the concentration-time profiles of GABA were presented as area under the curve (AUC), the AUC_0–4h_ and AUC_0–24h_ of a single dose and repeated doses were statistically significant compared to the placebo control (AUC_0–4h_: single dose vs. control = 1042.92 ± 221.95 vs. 48.47 ± 2.46 h·ng/ml, *p* < 0.001; repeated dose vs. control = 1246.54 ± 396.46 vs. 48.47 ± 2.46 h·ng/ml, *p* < 0.001. AUC_0–24h_: single dose vs. control = 1451.68 ± 243.12 vs. 272.85 ± 14.28 h·ng/ml, *p* < 0.001; repeated dose vs. control = 1778.69 ± 433.21 vs. 272.85 ± 14.28 h·ng/ml, *p* < 0.001). This indicates that GABA was rapidly absorbed, with maximum plasma concentrations achieved approximately 1–1.5 h after an oral dose, and subsequent mean elimination half-life in a range of 5–5.2 h. Additional derived pharmacokinetic parameters are summarized in Table 1. As shown, oral GABA administration both the single dose and repeated dose reached a comparable C_max_ (688 vs. 767 ng/ml), suggesting that GABA reached a C_max_ that was not dependent on the frequency of oral dosing. The cumulative coefficient was 1.11, which suggests almost no accumulation phenomenon during a course of oral GABA of 2 g administered three times per day for 7 consecutive days. There were no significant differences of the PK profiles observed between male and female volunteers.

**Table 1:** **Pharmacokinetics parameters**.

**Phase**	**T_max_(h)**	**C_max_(ng/ml)**	**t_1∕2_(h)**	**CL/F(L/h)**	**V/F (L)**	**AUC_0–24h_(h·ng/ml)**	**AUC_inf_(h·ng/ml)**	**RA**
Single-dose	1.5 (0.75–2)	688.53 ± 140.70%	5.08 ± 53.11%	2090.38 ± 85.17%	15924.60 ± 116.84%	932.91 ± 81.29%	956.77 ± 80.28%	
Repeated-dose	1 (0.75–2)	767.77 ± 224.25%	5.24 ± 24.31%	1723.03 ± 83.54%	19029.00 ± 123.72%	1078.11 ± 129.72%	1160.75 ± 121.64%	1.11

*Tmax is characterized with median and range, other parameters with geometric mean ± CV%*.

### Pharmacodynamic effects

**Figure 2:**
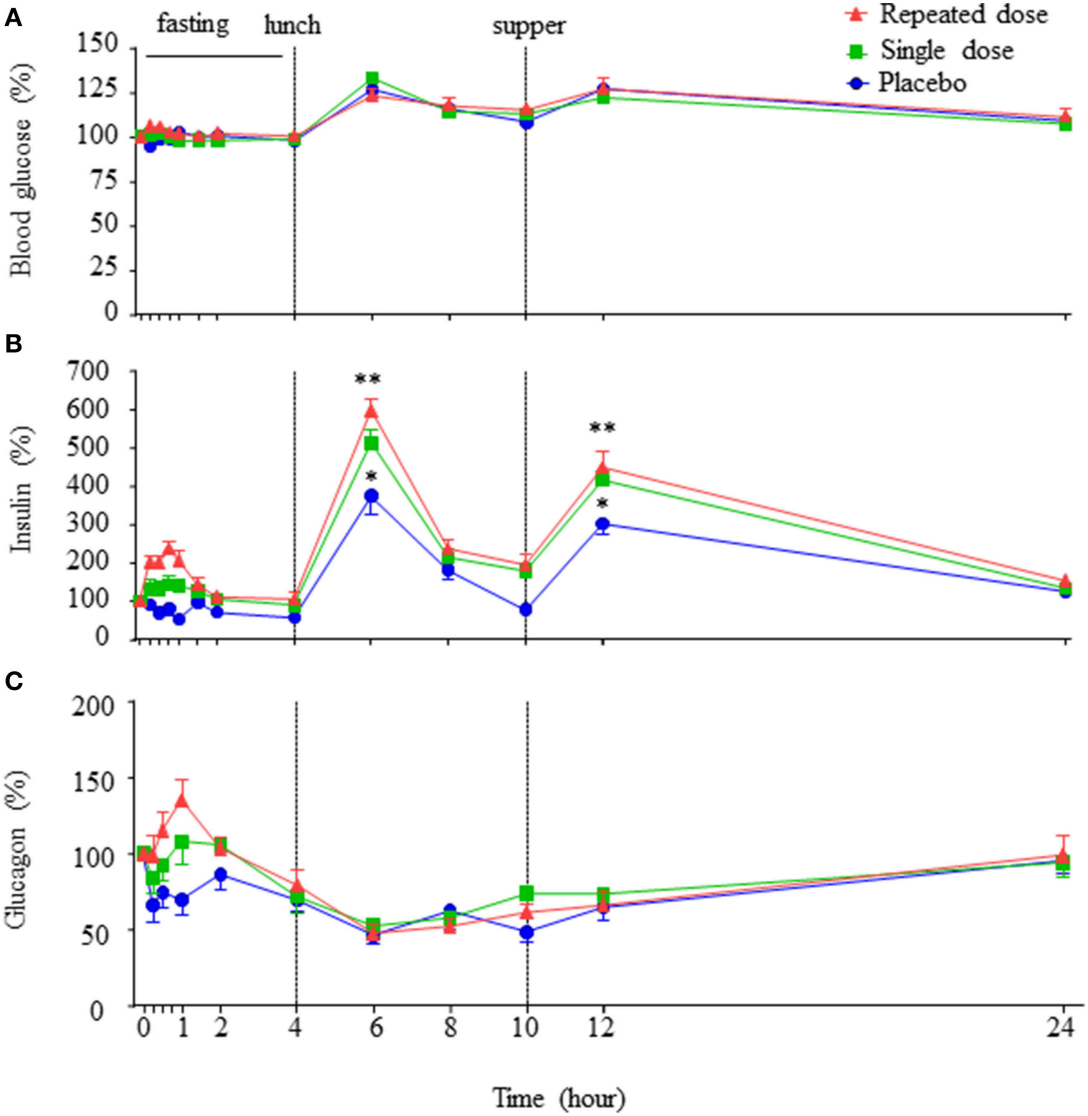
**Concentration-time profiles of blood glucose, insulin and glucagon**. The concentration-time curves of blood glucose, insulin, and glucagon in the three periods **(A–C)**, data are expressed as percent variation of baseline and presented as mean±SE. ^**^p < 0.01 vs. placebo. ^*^p < 0.05 vs. placebo.

There were no statistical differences in blood glucose, including the postprandial glucose levels, amongst the placebo, single dose, and repeated dose periods (Figure 2A), suggesting that oral GABA administration does not alter instantaneous blood glucose levels in healthy subjects, either under fasting or fed conditions.

Remarkably, corresponding to T_max_ of GABA, an increase in insulin levels was observed (Figure 2B). The insulin AUC_0–4h_ (fasting period) in the repeated dose period was higher than that of single dose and placebo periods, and insulin AUC_0–4h_ of single dose period was higher than that of placebo period (AUC_0–4h_: placebo vs. single dose = 94.91 ± 6.33 vs. 147.22 ± 13.95 h·pmol/L, *p* < 0.01; placebo vs. repeated dose = 94.91 ± 6.33 vs. 185.80 ± 12.82 h·pmol/L, *p* < 0.01; single dose vs. repeated dose = 147.22 ± 13.95 vs. 185.80 ± 12.82 h·pmol/L, *p* < 0.05). Furthermore, at the time points corresponding to meals (Figure 2B, 2 h after lunch, 2 h after supper), circulating insulin levels were higher in repeated dose and the single dose periods than that of the placebo period (2 h after lunch: repeated dose vs. placebo = 599.18 ± 32.46 vs. 375.71 ± 48.33%, *p* < 0.01; single dose vs. placebo = 514.23 ± 32.81 vs. 375.71 ± 48.33%, *p* < 0.05. Two hours after supper: repeated dose vs. placebo = 449.35 ± 42.66 vs. 304.17 ± 29.14%, *p* < 0.01; single dose vs. placebo = 415.47 ± 24.26 vs. 304.17 ± 29.14%, *p* < 0.05). In addition, insulin AUC_0–24h_ of both the repeated dose and single dose periods were all significantly higher than the placebo period (AUC_0–24h:_ repeated dose vs. placebo = 2230.91 ± 128.36 vs. 1478.74 ± 95.62 h·pmol/L, *p* < 0.01; single dose vs. placebo = 1986.54 ± 90.88 vs. 1478.74 ± 95.62 h·pmol/L, *p* < 0.01).

Interestingly, an increase in glucagon level corresponding to the T_max_ of GABA was also observed (Figure 2C). Particularly, the glucagon AUC_0–4h_ (fasting period) in the repeated dose period, as well as in the single dose period, were statistically higher than that of the placebo period (AUC_0–4h_: repeated dose vs. placebo = 65.46 ± 4.33 vs. 48.50 ± 4.83 h·pmol/L, *p* < 0.01; single dose vs. placebo = 59.71 ± 3.68 vs. 48.50 ± 4.83 h·pmol/L, *p* < 0.05). However, glucagon AUC_0–24h_ showed no difference among the three periods.

Notably, no significant difference of the insulin to glucagon ratio AUC_0–4h_ or AUC_0–24h_ was observed among the three periods. Similarly, there was no significant difference in the levels of active GLP-1 AUC_0–24h_ or AUC_0–4h_ noted among the three periods (Supplementary Figure 1).

While there was no significant difference of C-peptide AUC_0–24h_ among the three periods, there was a significant difference of AUC_0–4h_ between the repeated dose and placebo periods (Supplementary Figure 2, repeated dose vs. placebo = 8.83 ± 0.57 vs. 6.51 ± 0.58 h·pmol/L, *p* < 0.01).

**Figure 3:**
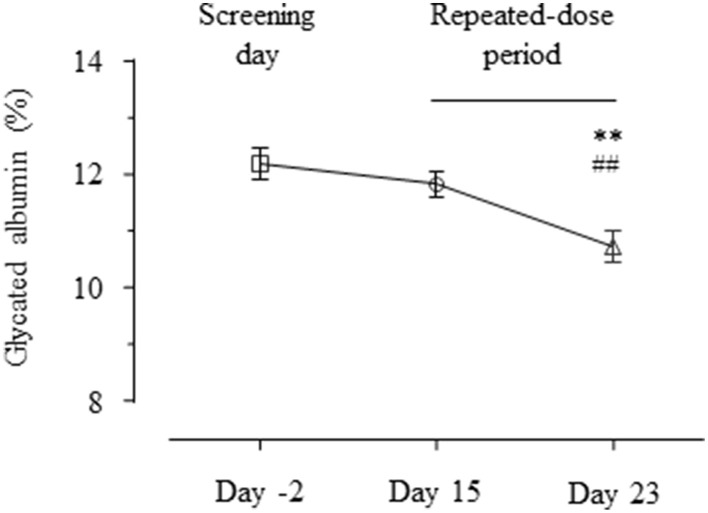
**Profiles of glycated albumin**. The glycated albumin levels on day 2, 15, and 23 are shown as mean ± SE. ^**^p < 0.01 vs. placebo; ^*##*^*p* < 0.01 vs. single dose.

In order to evaluate whether GABA exerts glycemic regulatory effects, we measured circulating levels of glycated albumin. The glycated albumin on day 23 was significantly lower than those on day 15 and day −2 (Figure 3, day 23 vs. day 15 = 10.73 ± 0.27 vs. 11.82 ± 0.23 %, *p* < 0.01; day 23 vs. day −2 = 10.73 ± 0.27 vs. 12.18 ± 0.26%, *p* < 0.01).

There were no significant differences in the PD parameters observed between male and female volunteers.

### Safety evaluation

Adverse events following GABA or placebo administration are presented in Table 2. The most frequently reported adverse events, which appeared to be related to the dosing regimens i.e., single dosing vs. repeated dosing were sore throat, throat burning, a skin burning sensation, headache, and dizziness. There were higher occurrences/frequencies of adverse events in the repeated dose period as compared to the single dose period, suggesting a dose, and duration dependency. However, there were no clinically relevant changes in all subjects in vital signs, ECG parameters, and physical examination. Safety laboratory measurements such as hematology, biochemistry, and urinalysis were all in normal ranges; except for four male volunteers that had slightly elevated alanine transaminase (ALT) and aspartate aminotransferase (AST) levels. This elevation of liver enzymes, however, was transient, without symptoms. Without changes of other biochemical indices of liver function such as bilirubin, in all cases, the ALT returned to the normal range without intervention at follow-up visit (Supplementary Table 2). There were no severe adverse events observed during the study.

**Table 2:** **Clinical and laboratory toxicity**.

	**Placebo period (D1–D2)**			**Single-dose period (D15–D16)**			**Repeated-dose period (D22–D30)**		
	***n***	***(%)***	***e***	***n***	***(%)***	***e***	***n***	***(%)***	***e***
Dry pharynx							4	36	7
Sore throat							8	73	22
Throat burning							4	36	12
Headache							5	45	8
Jaw pain							1	9	1
Neck pain							2	18	3
Dizziness				3	27	3	2	18	5
Poor sleep							4	36	4
Palpitation							2	18	2
Chest stuffiness							3	27	5
Fatigue							1	9	1
Nausea				1	9	1			
Skin needling sensation							3	27	3
Skin burning sensation							3	27	8
Limb numbness							4	36	9
Limb pain							1	9	1
Thirsty							1	9	1
ALT							4	36	8
AST							4	36	8

*n, Number of subjects; e, number of adverse events; %, proportion of subjects having the event*.

## Discussion

Preclinical studies by us and others reveal that GABA exerts anti-diabetic effects in several T1D mouse models due to its dual actions on β-cells and the immune system (Tian et al., 2004, 2013, 2014; Soltani et al., 2011; Purwana et al., 2014). This includes protection against β-cell apoptosis, β-cell regenerative effects, and anti-inflammatory effects. Importantly, GABA appears to act similarly on human and rodent cells, including xenotranplated human islet cells (Tian et al., 2013; Purwana et al., 2014). These studies suggest that GABA may be an effective therapeutic agent for treating T1D. However, there is little information available on GABA pharmacokinetics in humans, and to address this issue we investigated the effects of GABA in healthy volunteers. In this open-labeled, three-period, self-controlled study, we demonstrate that oral administration of GABA is effective and modulates circulating islet hormonal levels in healthy subjects.

We found that GABA reached peak concentration in the circulation after 1 ~ 1.5 h (Table 1) oral administration and remained increased for several hours. We observed that after oral GABA administration at a repeated daily dosing (2 g × 3) for consecutive 7 days, steady-state conditions appeared to be attained, which were accompanied by a 1/2 h shortened T_max_ and a higher C_max_ than that in the single dose period. As noted, previous studies (Petty et al., 1987) demonstrated that plasma GABA levels were not affected by gender, diet, exercise, and diurnal rhythm, which suggests that GABA has a reasonable stability for clinical pharmacological purposes. GABA is known to be metabolized by GABA transaminase enzyme (GABA + pyruvate = succinic semialdehyde + alanine; GABA + 2-oxoglutarate = succinic semialdehyde + glutamate; Bown and Shelp, 1997). However, these transamination products of GABA have no known pharmacological effects. There was nearly no accumulation noted after a three-daily dosing for a consecutive 7 days of oral administration, and with no severe adverse event observed in the healthy volunteers during the study course, it implies the possibility of long-term oral GABA clinical application.

GABA induced a dose-dependent increase in the fasting (8:00 AM–12:00 AM) and postprandial insulin secretion. GABA stimulated insulin secretion in humans, which is in accord with the higher c-peptide AUC_0–4h_ observed in the repeated dose period. Indeed, *in vitro* studies using isolated human islets recently showed that GABA dose-dependently increased insulin secretion in a GABA receptor dependent manner (Prud'homme et al., 2013). In accord with this, GABA induced β-cell membrane depolarization in rodent islets (Wendt et al., 2004; Soltani et al., 2011) and human islets (Braun et al., 2010) through A-type GABA receptor (GABAAR) dependent opening of the voltage dependent Ca^2+^ channel (Braun et al., 2010; Purwana et al., 2014). It induced membrane depolarizing effects in β-cells, which contrasts with its hyperpolarizing effects in the pancreatic α-cells (Rorsman et al., 1989; Xu et al., 2006).

Glucagon is an important hormone that counterbalances insulin actions on blood glucose homeostasis by stimulating hepatic glycogenolysis and gluconeogenesis (Gromada et al., 2007; Bansal and Wang, 2008). *In vitro* studies showed that while GABA enhances insulin secretion in β-cells, it suppresses glucagon release from the α-cells (Rorsman et al., 1989; Xu et al., 2006; Braun et al., 2010; Soltani et al., 2011; Purwana et al., 2014). However, in this study we found that GABA increases the circulating levels of both insulin and glucagon, and that the ratio of insulin to glucagon was not altered. The reason for this discrepancy between *in vitro* and *in vivo* results is unclear, and requires further investigation. However, we postulate that this represents a normal physiological response to control blood glucose levels. Indeed, blood glucose levels were not significantly changed in healthy subjects upon oral GABA administration under the fasting conditions, consistent with the notion that elevated glucagon levels countered the action of insulin.

GLP-1 is an important incretin hormone in the regulation of glucose homeostasis (Campbell and Drucker, 2013). We therefore examined if oral GABA administration affects circulating GLP-1 levels in the healthy subjects. Under fasting conditions, the circulating GLP-1 levels were relatively constant, and there were significant increase in postprandial active circulating GLP-1 levels (both lunch and supper) in the subjects. Notably, oral GABA administration (either single or repeated dose) had no significant effects on the active GLP-1. It is interesting to note that some previous *in vitro* studies suggest potential reciprocal interactions between GLP-1 and GABA (Gameiro et al., 2005; Wang et al., 2007), Nevertheless, our data suggested that at least under *in vivo* conditions, oral GABA administration at the present regime did not change active GLP-1 levels in healthy humans.

GABA significantly decreased glycated albumin levels after oral administration in repeated dosing (but not the single dose). Glycated albumin is an indicator of relatively recent (1–2 week) changes in blood glucose and thus has been considered as an indication of glycemic control (Inaba et al., 2007; Koga and Kasayama, 2010). Our results suggest that GABA administration might improve glycemic control. However, blood glucose levels monitored at least on day 1, 8, 22 (placebo, single dose, repeated dose) showed no changes between the three groups. Therefore, there is a possibility that GABA reduced glycated albumin levels through biochemical mechanisms other than blood glucose. Further studies are warranted to investigate the underlying mechanism of action.

There were no cases of hypoglycemia resulting from oral GABA administration in this study. Furthermore, although there were some transient adverse effects which appeared to be dose and duration dependency, there were no severe adverse events found during the entire course of study.

To our knowledge, this is the first clinical study investigating the pharmacokinetics of GABA in healthy subjects. A limitation was the lack of dose escalation to determine the optimal dose of GABA in humans. A future double-blind, randomized, placebo-controlled study is required to determine the optimal dose of GABA in humans, in either healthy or diabetic conditions.

In summary, orally administered GABA is rapidly absorbed by the gastrointestinal tract and remains elevated in the circulation for hours. It exerts stimulatory effects on insulin (and c-peptide) secretion, raising the possibility of its use in the treatment of diabetes. However, in healthy subjects it has no effects on instantaneous blood glucose levels. This may in part be attributed to GABA-induced counter regulatory mechanisms, especially elevated glucagon, which prevent hypoglycemia in the face of increased insulin levels. Our study was limited to healthy subjects, and the effects of GABA in diabetic patients may be different, and will be investigated in future studies. In terms of dosage, our findings support a three-daily dosing scheme. Since GABA targets diabetes in the key areas of islet-cell protection, regeneration, and immunotherapy, this study provides valid pharmacokinetics parameters to investigate its actions in diabetic subjects.

## Funding

This research is supported by grants from the Juvenile Diabetes Research Foundation (JDRF, grant number: JDRF17-2013-499). QW's current research is supported by JDRF (2015-64-Q-R), CDA(OG-3-13-4066-QW), NSFC(81570518, 81370877).

## Author contributions

ZJ, YL, and QW contributed to the conception and design of the research; JL, ZZ, XL, YW, FM, JM, XL, GC, JZ, ZJ, YL, and QW contributed to the acquisition of data; JL, ZZ, JM, JY, NZ, ZJ, and QW contributed to the analysis and interpretation of data; JL, DJ, YW, MA, DG, GP, ZJ, and QW contributed to drafting the article. All authors have revised the manuscript critically for important intellectual content and given final approval of the version to be published. QW is responsible for the integrity of the work as a whole.

### Conflict of interest statement

The authors declare that the research was conducted in the absence of any commercial or financial relationships that could be construed as a potential conflict of interest.

## Footnotes

^1^http://www.fda.gov/ucm/groups/fdagov-public/@fdagov-foods-gen/documents/document/ucm264254.pdf

## Supplementary material

Supplementary Figure 1 **Concentration-time profiles of GLP-1 (active form)**. The concentration-time curves of GLP-1 (active form) in the three periods, data are expressed as percent variation of baseline and presented as mean ± SE.

Supplementary Figure 2 **Concentration-time profiles of c-peptide**. The concentration-time curves of c-peptide in the three periods, data are expressed as percent variation of baseline and presented as mean ± SE.

Supplementary Table 1 **Trial Design**.

Supplementary Table 2 **ALT and AST elevation of four subjects during the repeated period**.

## Abbreviations

GABA: γ-aminobutyric acid
T1D: type 1 diabetes
C_max_: maximum concentration
PK: pharmacokinetics
PD: pharmacodynamics
FDA: Food and Drug Administration
T_max_: time to maximum plasma concentration
t1/2: terminal elimination half-life
CL/F: apparent clearance
V/F: apparent volume of distribution
AUC_0–4 h_: area under the curve from the time zero to 4 h
AUC_0–24 h_: area under the curve from the time zero to 24 h
AUC_0–t_: area under the curve from the time zero to the last quantifiable concentration
AUC_inf_: area under the curve from the time zero extrapolated to infinity
RA: ratio of accumulation
ALT: alanine transaminase
AST: aspartate aminotransferase
GLP-1: glucagon-like peptide-1.
