# Supplementary figures and images for: Study of GABA in Healthy Volunteers: Pharmacokinetics and Pharmacodynamics
Source: Front Pharmacol. 2015 Nov 10;6:260. doi: 10.3389/fphar.2015.00260 (PMC4639630; doi:10.3389/fphar.2015.00260)

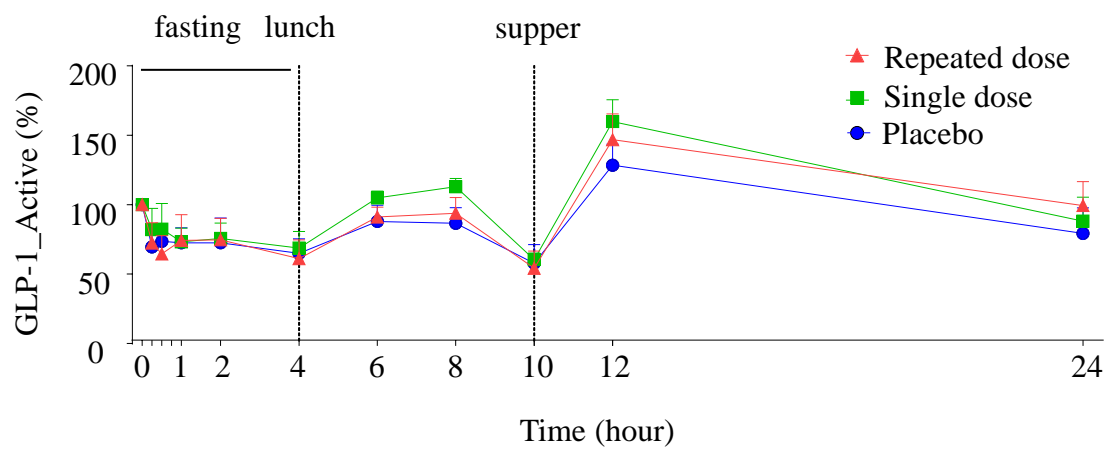

Supplement: Supplementary Figure 1 — Concentration-time profiles of GLP-1 (active form). The concentration-time curves of GLP-1 (active form) in the three periods, data are expressed as percent variation of baseline and presented as mean ± SE. [file Image1.PDF]

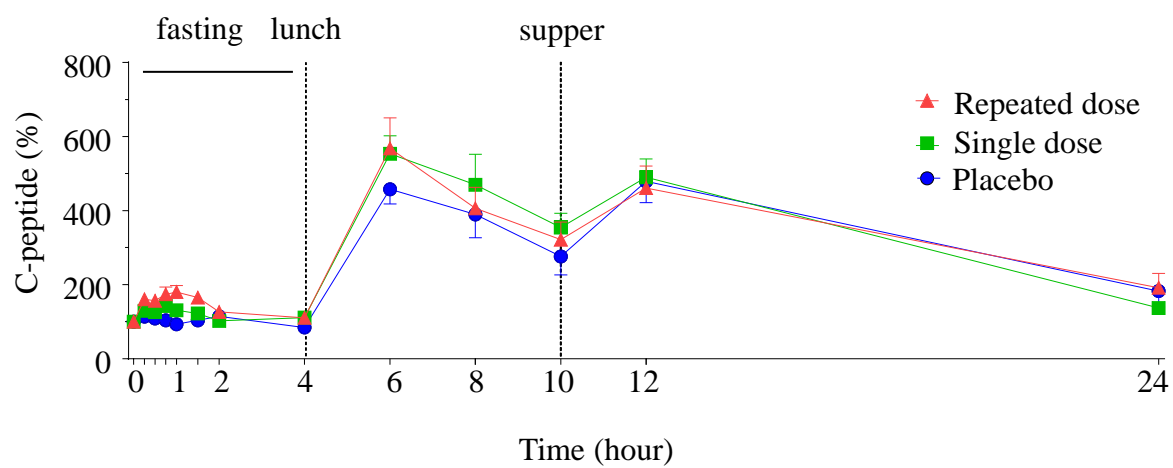

Supplement: Supplementary Figure 2 — Concentration-time profiles of c-peptide. The concentration-time curves of c-peptide in the three periods, data are expressed as percent variation of baseline and presented as mean ± SE. [file Image2.PDF]
