# Supplementary material for: Study of GABA in Healthy Volunteers: Pharmacokinetics and Pharmacodynamics
Source: Front Pharmacol. 2015 Nov 10;6:260. doi: 10.3389/fphar.2015.00260 (PMC4639630; doi:10.3389/fphar.2015.00260)
Supplement: Supplementary Table 1 — Trial Design. [file Table1.PDF]

Supplementary Table 1. Trial Design

	**Screening period**		**Placebo period**		**Follow-up period**					**Single-dose period**		**Follow-up period**					**Repeated-dose period**									**Follow-up period**		
**Time**	D-2	D-1	D1	D2	D3	D4	D5	D6	D7	D8	D9	D10	D11	D12	D13	D14	D15	D16	D17	D18	D19	D20	D21	D22	D23	D24	D25	D26
**Vital signs**	X	X	X	X	X	X	X	X	X	X	X	X	X	X	X	X	X	X	X	X	X	X	X	X	X	X	X	X
**Physical examination**	X								X	X														X				
**Drug administration**			qd							qd							tid	tid	tid	tid	tid	tid	tid	qd				
**ECG**	X	X	X	X						X	X	X	X				X						X	X	X	X	X	
**Hematology**	X			X						X	X						X							X	X			
**Blood chemistry**	X			X						X	X						X							X	X			
**HBs-Ag**	X																								X			
**HCV-Ab**	X																								X			
**HIV**	X																								X			
**Blood sugar**	X		X							X														X				
**Insulin**	X		X							X														X				
**C-peptide**	X		X							X														X				
**Glucagon**			X							X														X				
**GLP-1**			X							X														X				
**Urinalysis**	X			X						X	X						X							X	X			
**Urine microscopic evaluation**	X			X						X	X						X							X	X			
**Urine pregnancy test**	X																								X			
**Glycated Albumin**	X																X								X			
**GABA**			X							X														X				

Drug administration, qd: 2g/day; tid: 2g×3/day
Time: D, day
