# Supplementary material for: Study of GABA in Healthy Volunteers: Pharmacokinetics and Pharmacodynamics
Source: Front Pharmacol. 2015 Nov 10;6:260. doi: 10.3389/fphar.2015.00260 (PMC4639630; doi:10.3389/fphar.2015.00260)
Supplement: Supplementary Table 2 — ALT and AST elevation of four subjects during the repeated period. [file Table2.PDF]

Supplementary Table 2. ALT and AST elevation of 4 subjects during the repeated period

Subject No.	Repeated-dose period (D22, D23)		Follow-up period (D37)	
	ALT	AST	ALT	AST
1	172, 157	43, 48	26	7
3	56, 90	57, 51	18	11
4	186, 166	54, 46	13	12
5	72, 77	60, 53	21	9

Normal range: ALT，0-45 U/L；AST，0-40 U/L
D1 (day 1) was the day when placebo tablets were taken
